# The *VEGF *-634G>C promoter polymorphism is associated with risk of gastric cancer

**DOI:** 10.1186/1471-230X-9-77

**Published:** 2009-10-16

**Authors:** Xiaoxiang Guan, Hui Zhao, Jiangong Niu, Dongfeng Tang, Jaffer A Ajani, Qingyi Wei

**Affiliations:** 1 Department of Epidemiology, the University of Texas M. D. Anderson Cancer Center, Houston, Texas 77030, USA; 2 Department of Pathology, the University of Texas M. D. Anderson Cancer Center, Houston, Texas 77030, USA; 3 Department of Gastrointestinal Medical Oncology, the University of Texas M. D. Anderson Cancer Center, Houston, Texas 77030, USA

## Abstract

**Background:**

Both TGF-β1 and VEGF play a critic role in the multiple-step process of tumorgenesis of gastric cancer. Single nucleotide polymorphisms (SNPs) of the *TGFB1 *and *VEGF *genes have been associated with risk and progression of many cancers. In this study, we investigated the association between potentially functional SNPs of these two genes and risk of gastric cancer in a US population.

**Methods:**

The risk associated with genotypes and haplotypes of four *TGFB1 *SNPs and four *VEGF *SNPs were determined by multivariate logistic regression analysis in 171 patients with gastric cancer and 353 cancer-free controls frequency-matched by age, sex and ethnicity.

**Results:**

Compared with the *VEGF*-634GG genotype, the -634CG genotype and the combined -634CG+CC genotypes were associated with a significantly elevated risk of gastric cancer (adjusted OR = 1.88, 95% CI = 1.24-2.86 and adjusted OR = 1.56, 95% CI = 1.07-2.27, respectively). However, none of other *TGFB1 *and *VEGF *SNPs was associated with risk of gastric cancer.

**Conclusion:**

Our data suggested that the *VEGF*-634G>C SNP may be a marker for susceptibility to gastric cancer, and this finding needs to be validated in larger studies.

## Background

Gastric cancer is one of the major causes of cancer-related deaths worldwide, although both incidence and mortality of gastric cancer have been declining in recent years [1]. In 2008, there were 21,500 new gastric cancer patients, and 10,880 patients died of this disease in the United States [2]. Epidemiological studies have identified many risk factors for gastric cancer, including older age, being a man, Helicobacter pylori (H. pylori) bacteria infection, diets high in smoked foods, salted fish and meats, and pickled vegetables, tobacco use and obesity [2]. Although about two-thirds of gastric cancer could be prevented by changing lifestyle and diet habits [3], the fact that some individuals develop gastric cancer while others do not when having similar exposures suggests that genetic factors may also play an important role in the etiology of gastric cancer.

It has been reported that both TGF-β1 and VEGF played an important role in the oncogenesis of gastric cancer [4,5]. Both *TGFB1 *and *VEGF *genes are highly polymorphic, reportedly having 168 and 140 variants, respectively, but few are within the promoters or coding regions that may be potentially functional http://www.ncbi.nlm.nih.gov/SNP/. Of these variants, several common single nucleotide polymorphisms (SNPs) have been described as important [6-8] and reported to be involved in the etiology of various cancers [9-12]. For example, some *TGFB1 *and *VEGF *variants are associated with protein functions, which may contribute to an individual's susceptibility to cancer [13]. Several studies have investigated the association between *TGFB1 *and *VEGF *SNPs and risk of cancers, including breast cancer [14-16], lung cancer [17,18], and gastric cancer [19-22], but the results were inconsistent.

To investigate the role of *TGFB1 *and *VEGF *SNPs in the etiology of gastric cancer, we conducted a case-control study to evaluate the association between three *TGFB1 *SNPs (one promoter SNP -509C>T and two exon 1 SNPs +869T>C and +915G>C) and three *VEGF *SNPs (one promoter SNP -1498T>C, one 5'-untranslated region SNP -634G>C and one 3'-untranslated region SNP +936C>T) and gastric cancer risk in a US population.

## Methods

### Study population

The case-control study consisted of 171 patients with newly diagnosed and histologically confirmed gastric cancer at The University of Texas MD Anderson Cancer Center (Houston, TX) between February 1990 and July 2008. The 353 cancer-free controls subjects were selected from an ongoing molecular epidemiology study of the head and neck cancer since 1995 [23] by frequency matching by age, sex and ethnicity. All cancer-free control subjects were recruited from persons who were not hospital patients, nor seeking health care but accompanying the patients visiting the clinics, and who were genetically unrelated to the cases. The study protocol and informed consent form used in this study were approved by our institutional review board.

### Genotyping

Genomic DNA was extracted from the buffy coat fraction of each blood sample by using a Blood Mini Kit (Qiagen, Valencia, CA) according to the manufacturer's instructions. DNA purity and concentrations were determined by spectrophotometric measurement of absorbance at 260 and 280 nm by UV spectrophotometer.

The selected three *TGFB1 *SNPs [-509C>T (rs1800469) in the promoter, +869T>C (rs1800470) and +915G>C (rs1800471) in the exon 1] and three promoter *VEGF *SNPs [-1498T>C (rs833061) in the promoter and -634G>C (rs2010963) in the 5'-untranslated region, and *VEGF *+936C>T (rs3025039) in the 3'-untranslated region] were genotyped using polymerase chain reaction-restriction fragment length polymorphism (PCR-RFLP) method. These six SNPs are relatively common (a minor allele frequency > 0.05) in Caucasian populations. Genotypes of *TGFB1 *SNPs were determined as previously described [24]. Assays on the *VEGF *-1498T>C (rs833061), -634G>C (rs2010963), and +936C>T (rs3025039) SNPs were previously reported [17]

For the PCR-RFLP assay, the genotyping was performed without knowing the subjects' case and control status, and almost the same number of cases and controls were assayed in each 96-well PCR plate. Two research assistants independently read the gel pictures and performed the repeated assays, if they did not reach a consensus on the tested genotype. In addition, 10% of the samples were randomly selected to perform the repeated assays, and the results were 100% concordant.

### Statistical Analysis

The χ^2 ^tests were used to compare the distribution of demographic variables and selected risk factors between cases and controls. The Hardy-Weinberg equilibrium (p^2^+2pq+q^2 ^= 1), where p is the frequency of the variant allele and q = 1-p, was tested by a goodness-of-fit χ^2 ^test to compare the observed genotype frequencies with the expected genotype frequencies in cancer-free controls. Unconditional logistical regression analysis was used to calculate odds ratios (ORs) and their 95% confidence intervals (CIs) with and without adjustment for age, sex, ethnicity, smoking status and alcohol use. Age was also dichotomized according to the median age (57 years) of the controls. The logistic regression model was also used for the trend test of the genotypes with different number of the minor alleles. The 2-linkage disequilibrium (LD) program and Proc ALLELE in SAS/Genetics software were used to detect the LD of any pair of SNPs. Proc HAPLOTYPE in SAS/Genetics software using the expectation-maximization (EM) algorithm was applied to generate maximum likelihood estimates of haplotype frequencies. Haplotype were combined when their frequency was <5%. The joint effects of the *TGFB1 *and *VEGF *SNPs and their interactions with smoking and drinking on gastric cancer risk were also evaluated. Finally, we calculated the false-positive report probability (FPRP) to test for false-positive associations, using prior probabilities of 0.0001, 0.001, 0.01, 0.1, and 0.25. The OR was set close to the observed value obtained in our study, and a probability of <0.2 was considered a noteworthy association. All statistical tests were two-sided, with a *P *value of 0.05 considered significant, by using SAS software (version 9.1; SAS Institute, Cary, NC).

## Results

### Characteristics of the study population

This analysis included 171 gastric cancer cases and 353 cancer-free controls. Because we used frequency matching, there were no significant difference in distributions of age, sex and ethnicity between the cases and controls (*P *= 0.135, 0.444 and 0.486, respectively). For the tumor types, more than 50% of the cases had intestinal type, about 125 had difuse type, and the remaining was mixed. There were more current and former smokers in cases (19.9% and 39.2%, respectively) than in controls (13.6% and 38.5%, respectively), but the difference was not statistically significant (*P *= 0.126). However, there were significantly more alcohol drinkers among cases than among controls (*P *= 0.037) (Table 1). These variables were further adjusted for in multivariate logistic regression models to control for any residual effect of their confounding on the main effect of selected SNPs.

**Table 1:** Frequency distributions of selected variables in gastric cancer cases and cancer free controls

	Cases (n = 171)		Controls (n = 353)		
Variables	n	%	n	%	P*
Age (years)	59.7 ± 12.6		57.6 ± 11.2		0.135
≤ 45	24	14.0	47	13.3	
45-59	90	52.6	216	61.2	
>59	57	33.3	90	25.5	
Sex					
Female	56	32.7	104	29.5	0.444
Male	115	67.3	249	70.5	
Ethnicity					
Non-Hispanic White	120	70.2	258	73.1	0.486
Non-White	51	29.8	95	26.9	
Smoking status					
Current	34	19.9	48	13.6	
Former	64	39.2	136	38.5	0.126
Never	70	40.9	169	47.9	
Alcohol use					
Current	63	36.8	115	32.5	
Former	33	19.3	45	12.8	0.037
Never	75	43.9	193	54.7	
Tumor type					
Intestinal	101	59.1			
Diffuse	20	11.7			
Mixed	50	29.2			

* Two-sided χ^2 ^test.

### TGFB1 and VEGF genotypes and risk of gastric cancer

The genotype and allele distributions of the three *TGFB1 *SNPs and three *VEGF *SNPs between the cases and controls are summarized in Table 2. The observed genotype frequencies of these SNPs were all in agreement with the Hardy-Weinberg equilibrium in the control subjects, except for *TGFB1 *-509C>T and *VEGF *-634G>C, indicating some possible selection bias in the hospital-based controls. Although none of the minor alleles had a different frequency distribution between the cases and the controls, the *VEGF*-634G>C genotype frequencies were statistically significantly between the cases (40.4% for GG, 42.1% for CG, and 17.5% for CC) and the controls (51.0% for GG, 28.0% for CG and 21.0% for CC) (*P *= 0.003) (Table 2).

**Table 2:** Genotype frequencies of *TGFB1 *and *VEGF *polymorphism gastric cancer cases and controls and their associations with risk of gastric cancer

	Cases (n = 171)		Controls (n = 353)					
						
Genotypes	n	%	n	%	***P***^†^	Crude OR (95% CI)	Adjusted OR (95% CI) *	***P***^‡^
*TGFB1 -509 C >T (rs1800469)*					0.404			
CC	125	73.1	238	67.4		1.00	1.00	
CT	33	19.3	80	22.7		0.79 (0.50-1.24)	0.81 (0.51-1.30)	0.386
TT	13	7.6	35	9.9		0.71 (0.36-1.39)	0.71 (0.36-1.40)	0.323
CT+TT	46	26.9	115	32.6		0.76 (0.51-1.14)	0.78 (0.52-1.18)	0.239
C allele	0.173		0.212		0.246**			
*TGFB1 869 T >C (rs1800470)*					0.805			
TT	57	33.3	111	31.4		1.00	1.00	
CT	83	48.5	170	48.2		0.95 (0.63-1.44)	1.01 (0.67-1.55)	0.947
CC	31	18.1	72	20.4		0.84 (0.49-1.42)	0.81 (0.48-1.39)	0.448
CT+CC	114	66.7	242	68.6		1.16 (0.73-1.85)	0.92 (0.62-1.35)	0.374
C allele	0.424		0.445		0.727**			
*TGFB1 915 G >C (rs1800471)*					0.447			
GG	151	88.3	313	88.7		1.00	1.00	
CG	18	10.5	39	11.0		0.96 (0.53-1.73)	0.99 (0.54-1.83)	0.994
CC	2	1.2	1	0.3		4.15 (0.37-46.1)	4.91 (0.43-56.3)	0.201
CG+CC	20	11.7	40	11.3		1.04 (0.59-1.83)	1.09 (0.56-1.96)	0.776
C allele	0.064		0.058		0.715**			
*VEGF -1498T > C (rs833061)*					0.748			
TT	51	29.8	111	31.4		1.00	1.00	
CT	83	48.6	159	45.0		1.14 (0.74-1.74)	1.12 (0.73-1.74)	0.597
CC	37	21.6	83	23.6		0.97 (0.58-1.62)	0.99 (0.59-1.67)	0.973
CC+CT	120	70.2	242	68.6		1.08 (0.73-1.61)	1.08 (0.72-1.62)	0.715
C allele	0.459		0.460		0.984**			
*VEGF -634G > C (rs2010963)*					0.006			
GG	69	40.4	51.0			1.00	1.00	
CG	72	42.1	28.0			**1.90 (1.26-2.86)**	**1.88 (1.24-2.86)**	**0.003**
CC	30	17.5	21.0			1.06 (0.64-1.76)	1.10 (0.66-1.85)	0.712
CG+CC	102	59.6	49.0			**1.54 (1.06-2.23)**	**1.56 (1.07-2.27)**	**0.022**
C allele	0.386		0.349		0.491**			
*VEGF +936C > T (rs3025039)*					0.550			
CC	127	74.3	78.2			1.00	1.00	
CT	41	24.0	19.8			1.27 (0.82-1.97)	1.29 (0.83-2.02)	0.262
TT	3	1.8	2.0			0.93 (0.24-3.66)	1.12 (0.28-4.58)	0.837
CC+CT	44	25.8	21.8			1.24 (0.81-1.90)	1.28 (0.83-1.97)	0.269
T allele	0.137		0.119		0.481**			

* Adjusted for age, gender, ethnicity, smoking status and alcohol status in a logistic regression model.

^†^Two-sided χ ^2 ^test for either genotype distribution or allele frequency.

^‡ ^Two-sided χ ^2 ^test for difference in frequency distribution of genotype between cases and controls by adjusted for age, gender, ethnicity, smoking status and alcohol status.

** Two-sided χ ^2 ^tests for the differences in the minor allele frequencies between cases and controls

Further logistic regression analysis revealed that, of the six SNPs examined, only the *VEGF *-634CG heterozygous and the combined -634CG+CC variant genotypes were associated with a significantly elevated risk of gastric cancer (adjusted OR = 1.88, 95% CI = 1.24-2.86 and adjusted OR = 1.56, 95% CI = 1.07-2.27, respectively). In further stratified analysis by age, sex, ethnicity, alcohol use and smoking status, the risk associated with the combined -634CG+CC variant genotypes was more evident in subgroups of ever smokers (adjusted OR = 1.75, 95% CI = 1.06-2.90), never drinker (adjusted OR = 2.00, 95% CI = 1.14-3.54) and non-white subjects (adjusted OR = 2.39, 95% CI = 1.15-4.98) (Table 3). This kind of significant association was not found for the three *TGFB1 *SNPs and other two *VEGF *SNPs (Table 2).

**Table 3:** Stratification analysis of gastric cancer risk associated with the *VEGF -634 G>C *genotype frequencies

	**Cases (n = 171)**		**Controls (n = 353)**		Adjusted OR (95% CI)*		
				
**Variables**	**GG** **N (%)**	**CG+CC** **N (%)**	**GG** **N (%)**	**CG+CC** **N (%)**	**GG**	**CG+CC**	***P***^†^
			
Age (years)							
≤ 57	30 (42.3)	41 (57.7)	95 (52.8)	85 (47.2)	1.00	1.50 (0.84-2.64)	0.160
> 57	39 (39.0)	61 (61.0)	85 (49.1)	88 (50.9)	1.00	1.58 (0.95-2.63)	0.080
Sex							
Female	18 (32.1)	38 (67.9)	49 (47.1)	55 (52.9)	1.00	1.86 (0.94-3.69)	0.075
Male	51 (44.4)	64 (55.6)	131 (52.6)	118 (47.4)	1.00	1.44 (0.91-2.28)	0.116
Ethnicity							
Hispanic White	53 (44.2)	67 (55.8)	129 (50.0)	129 (50.0)	1.00	1.32 (0.85-2.06)	0.219
Non-white	16 (31.4)	35 (68.6)	51 (53.7)	44 (46.3)	1.00	**2.39 (1.15-4.98)**	**0.020**
Smoking status							
Never	27 (38.6)	43 (61.4)	79 (46.7)	90 (53.3)	1.00	1.33 (0.75-2.38)	0.336
Ever	42 (41.6)	59(58.4)	101 (54.9)	83 (45.1)	1.00	**1.74 (1.06-2.90)**	**0.032**
Drinking status							
Never	25 (33.3)	50 (66.7)	99 (51.3)	94 (48.7)	1.00	**2.00 (1.14-3.54)**	**0.017**
Ever	44 (45.8)	52 (54.2)	81 (50.6)	79 (49.4)	1.00	1.24 (0.74-2.07)	0.411

* Adjusted for age, sex, ethnicity, smoking status and alcohol status in a logistic regression model where it was appropriate.

^†^Two-sided χ ^2 ^test for the ORs obtained from the multivariate logistic regression with adjustment for age, sex, ethnicity, smoking status and alcohol status

### TGFB1 and VEGF haplotypes and risk of gastric cancer

To further analyze possible combined effects of the *TGFB1 *and *VEGF *SNPs, we estimated the haplotypes on the basis of the observed genotypes. As shown in Table 4, the four common *TGFB1 *haplotypes (CTG, CCG, TCG and CCC) represented 85.6% and 89.9% of the chromosomes of the controls and cases, respectively. For the *VEGF *haplotypes, the common alleles included CGC, TCC, CCC and CGT. However, the frequency distributions of these haplotypes between the cases and controls was not significantly different for both *TGFB1 *(*P *= 0.759) and *VEGF *(*P *= 0.808), nor were they associated with risk of gastric cancer.

**Table 4:** Frequency distribution of the *TGFB1 *and *VEGF *haplotype alleles between gastric cancer cases and controls and their associations with risk of Gastric Cancer

	Cases		Controls			
				
Genotypes	n	%	n	%	Crude OR (95% CI) *	P**
*TGFB1 *alleles	(No. of variant alleles)^†^					
C-T-G	194	46.0	387	40.0	1.29 (0.12-13.7)	0.830
C-C-G	107	25.4	216	24.6	1.26 (0.11-13.9)	0.851
T-C-G	56	13.3	144	16.4	1.93 (0.17-21.8)	0.594
C-C-C	22	5.21	40	4.6	0.87 (0.06-13.6)	0.919
*VEGF *alleles	(No. of variant alleles)^‡^					
C-G-C	135	24.6	278	30.0	0.56 (0.02-18.0)	0.744
T-C-C	115	20.9	200	21.6	0.42 (0.02-13.6)	0.621
C-C-C	65	11.8	90	9.7	0.46 (0.01-19.9)	0.686
C-G-T	35	6.4	45	4.9	0.25 (0.01-12.2)	0.482

* Calculated by using the most common haplotype as the reference. Ors represent the risk per copy of each haplotype.

† The number of *TGFB1 *haplotype alleles; the variant (risk) alleles used for calculation were-509T, 869C or 915C.

‡ The number of *VEGF *haplotype alleles; the variant (risk) alleles used for calculation were -1498C, -634C or 936T.

**Two-sided χ ^2 ^test for the ORs obtained from the multivariate logistic regression with adjustment for age, sex, ethnicity, smoking status and alcohol status

Finally, because the sample size of this study was small, we calculated FPRP for the positive findings. We found that under the assumption of a prior probability of 0.1, the FPRP of *VEGF *-634CG vs. -634GG for yielded values of 0.13, suggesting that the *VEGF *-634CG vs. -634GG findings was noteworthy. However, the FPRPs of *VEGF *-634CG+CC vs. -634GG for all subjects, non-whites, ever smokers and never drinkers yielded values of 0.31, 0.54, 0.49 and 0.38, respectively, which were likely to be false positive findings. Therefore, our findingds need to be validated in larger studies.

## Discussion

Gastric cancer is a genetic disease developing from a multifactorial, multigenetic and multistage process [25,26]. It was widely accepted that both genetic and environmental factors may be involved in the etiology of gastric cancer [27]. During the multistage carcinogenesis, *TGFB1 *may be involved in multiple important cellular processes, play a biphasic role in carcinogenesis, and inhibit proliferation of tumors in early stages of cancers, whereas it may also promote tumor growth and metastasis in later stages of cancers [28,29]. Several molecular epidemiological studies have shown an association of *TGFB1 *SNPs with risk of various cancers, including breast [14,15,30,31], prostate [32,33] and hepatocellular carcinoma [34,35] and gastric cancer [4,19].

The roles of -509C>T, +869T>C and +915G>C SNPs in *TGFB1 *have been implicated in the etiology of various human cancers. For example, in high-risk Chinese populations, *TGFB1 *-509C>T was associated with a reduced risk of gastric cancer [19] and the -509T variant genotypes were found to be associated with a decreased risk of among stage I+II cases [20]. In the present study, we did not observe a significant association between *TGFB1 *SNPs and gastric cancer risk, partly because our study size was small. Another possible explanation is that there may be an ethnicity difference in the etiology of gastric cancer. For example, the H. pylori infection was common in Asians but not in Caucasians [36], which may interact with genetic factors. Furthermore, cancer is a disease involving multiple genes/SNPs, and therefore the combined analysis of multiple gene or haplotype-based approach might be more powerful than the analysis of single allele or locus effect [19], which requires studies with much large sample sizes. It is also likely that our results could be biased owing to possible selection biases in hospital-based case-control studies. Therefore, larger studies are warranted to further test whether the *TGFB1 *variants are associated with gastric cancer risk.

Among other genetic factors, *VEGF*, the key mediator of angiogenesis, also plays an important role in the development of different kind of tumors, including gastric cancer. Several studies have investigated the associations between *VEGF *SNPs and risk of cancers, including breast, lung and gastric cancers [16,17,21,22,37], However, the results from published studies remain inconsistent rather than conclusive. Recently, Ke Q et al [21] found that none of four potentially functional SNPs (-2578C>A, -1498T>C, -634G>C, and +936C>T) of the *VEGF *gene or their haplotypes achieved a significant difference in their distributions between gastric cancer cases and controls in a Chinese population, whereas in a Greek study of the association between these four potentially functional SNPs in *VEGF *and gastric cancer risk in 100 cases and 100 controls [38], it was found that the -634CC variant genotype was associated with an increased the risk of gastric cancer with a marginal significance. However, the -634CC was shown to be associated with a significantly decreased risk of gastric cancer in a Korean population [22]. Our current study indicated that the heterozygous -634CG and the combined -634CG+CC carriers had an increased risk of gastric cancer when compared with the -634GG genotype. Again, these studies demonstrated some possible ethnic difference in the etiology of gastric cancer [19].

An obvious limitation in the present study was the lack of information on the H. pylori infection status of the subjects. Because H. pylori infection was relatively rare in the US compared with Asian countries, these patients were not tested for H. pylori infection upon their visit to the hospital. This defect of the study has to be corrected in future well-planned studies. Also, we observed that genetic effects were more profound in never drinkers in this study population, a phenomenon we often observed in our association studies, in which the risk associated with adverse genotypes appears to be higher in non-exposed or less exposed subjects, suggesting that the related susceptibility to cancer may be high in such subgroups. Alternatively, there may be some unknown risk factors that were not identified in these subgroups.

## Conclusion

In this hospital-based case-control study of sporadic gastric cancer, our finding supports the notion that variant genotypes of the *VEGF*-634C>G polymorphism, but not other tested SNPs, may contribute to gastric cancer risk. However, because we used a hospital-based case-control study design, the observed associations may have been biased or simply due to chances. Therefore, larger, prospective studies, particularly with the information about H. pylori infection, are needed to verify our findings.

## Abbreviations

***TGFB1***: Transforming growth factor beta 1; ***VEGF***: Vascular endothelial growth factor; **LD**: linkage disequilibrium; **PCR-RFLP**: polymerase chain reaction-restriction fragment length polymorphism; **OR**: odds ratio; **CI**: confidence interval; **SNP**: single nucleotide polymorphism.

## Competing interests

The authors declare that they have no competing interests.

## Authors' contributions

XG performed the genotyping work; XG, HZ, JN performed data analysis; XG, DT, QW participated in the design of the study; XG, QW wrote the manuscript; HZ, JN, DT and JA read and approved the manuscript; All authors read and approved the final manuscript for submission.

## Pre-publication history

The pre-publication history for this paper can be accessed here:

http://www.biomedcentral.com/1471-230X/9/77/prepub
